# Cellular response to DNA interstrand crosslinks: the Fanconi anemia pathway

**DOI:** 10.1007/s00018-016-2218-x

**Published:** 2016-04-19

**Authors:** David Lopez-Martinez, Chih-Chao Liang, Martin A. Cohn

**Affiliations:** Department of Biochemistry, University of Oxford, South Parks Road, Oxford, OX1 3QU UK

**Keywords:** DNA repair, Genomic instability, Phosphorylation, Ubiquitination, SUMO, FANCD2, FANCI, UHRF1

## Abstract

Interstrand crosslinks (ICLs) are a highly toxic form of DNA damage. ICLs can interfere with vital biological processes requiring separation of the two DNA strands, such as replication and transcription. If ICLs are left unrepaired, it can lead to mutations, chromosome breakage and mitotic catastrophe. The Fanconi anemia (FA) pathway can repair this type of DNA lesion, ensuring genomic stability. In this review, we will provide an overview of the cellular response to ICLs. First, we will discuss the origin of ICLs, comparing various endogenous and exogenous sources. Second, we will describe FA proteins as well as FA-related proteins involved in ICL repair, and the post-translational modifications that regulate these proteins. Finally, we will review the process of how ICLs are repaired by both replication-dependent and replication-independent mechanisms.

## Introduction

Our genome is constantly exposed to damage caused by both endogenous and exogenous sources. ICLs (interstrand crosslinks) are one of the most cytotoxic lesions because the two Watson and Crick strands of DNA are covalently bound together, causing an obstacle to replication and transcription. ICLs left unrepaired can lead to mutations, chromosome breakage, chromosome missegregation and mitotic catastrophe. To protect the genome from this type of lesion, the cells count on a highly complex repair pathway to detect the lesion, activate the cell cycle checkpoint and repair the ICLs. ICLs can be generated by naturally occurring compounds, such as psoralen and mitomycin C, as well as by chemically synthesized crosslinking agents, such as cisplatin. ICL-forming drugs are widely used as chemotherapeutic drugs against cancer. Reactive aldehydes have been shown to be one of the endogenous sources causing crosslinks. The chemical structure of the resulting ICL depends on the crosslinking agent implicated, and these different structures will lead to different cellular responses. The response to ICLs triggers a complex DDR (DNA damage response) including the Fanconi anemia (FA) pathway as well as the ATR (ATM and Rad3-related)/Chk1 pathway. Therefore, along the FA pathway, several signal transduction events take place mediated by multiple modifications including phosphorylation, ubiquitination and SUMOylation (small ubiquitin-like modifier) events. In order to fully repair the ICL, the FA pathway coordinates different processes including translesion synthesis (TLS), homologous recombination (HR) and nucleotide excision repair (NER). Therefore, there is extensive crosstalk between different DNA repair pathways during ICL repair.

## Origin of ICLs

ICLs are formed when the two strands of DNA are covalently bound together through a linker molecule commonly known as a crosslinking agent. The first crosslinking agents to be identified were the nitrogen mustards developed during the warfare of the early twentieth century. A better application for these compounds was found as chemotherapeutic agents, though their mechanism of action was still unknown. Later other compounds such as mitomycin C and cisplatin joined them as chemotherapeutic agents and they were all found to be crosslinking agents [1]. They can react with DNA and give rise to different kinds of products including DNA monoadducts, intrastrand crosslinks and ICLs with variable efficiencies. They also differ in their base specificity for ICL formation and the degree of distortion in the DNA double helix they generate. Some of these compounds, such as psoralens, which are produced by certain plants, may play an important role as environmental sources of ICLs. However, the search for endogenous sources of ICLs has rendered very interesting results in the past few years, suggesting that reactive aldehydes are one of the endogenous crosslinking agents [2]. Some candidates include products derived from lipid peroxidation such as malondialdehyde and crotonaldehyde, but also nitric oxide has been proposed [3].

### Nitrogen mustards

**Fig. 1:**
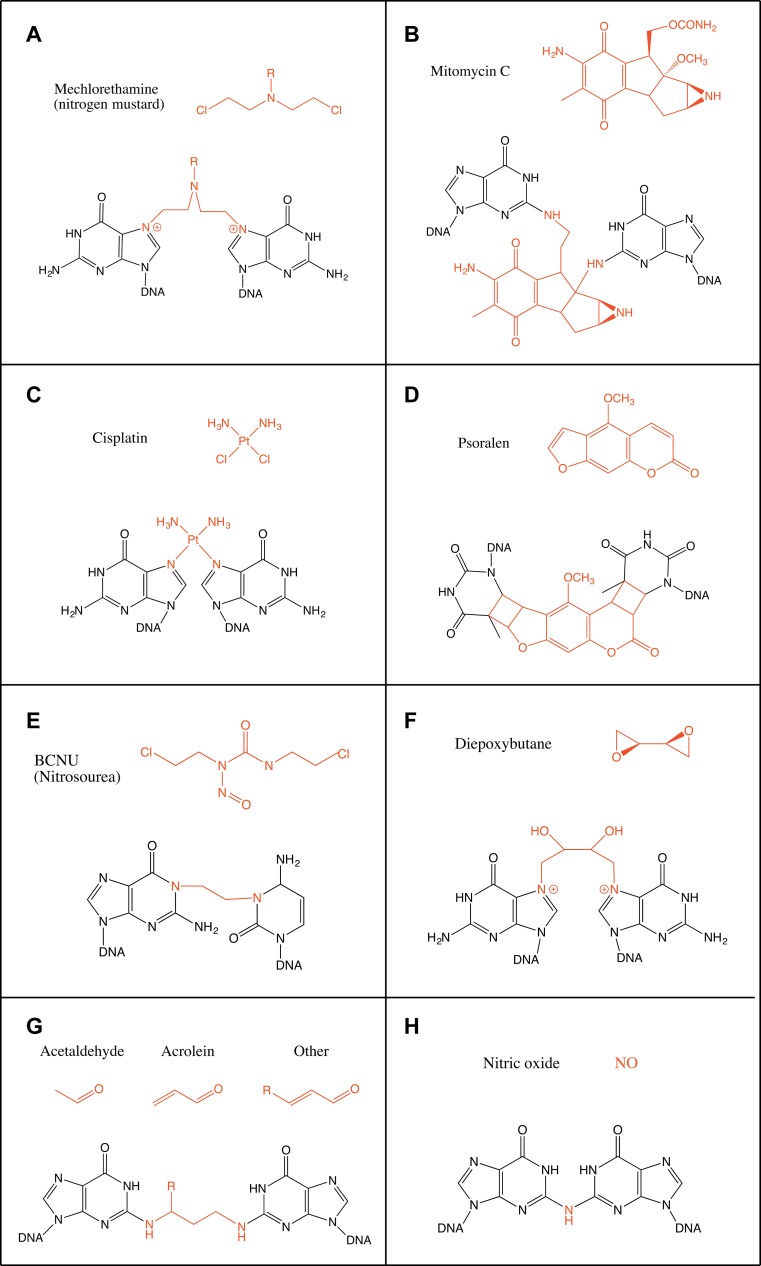
Schematic representation of the chemical structure of the main crosslinking agents and the ICLs they form. **a** Mechlorethamine (nitrogen mustard), **b** mitomycin C, **c** cisplatin, **d** psoralen, **e** BCNU (nitrosourea), **f** diepoxybutane, **g** aldehydes [acetaldehyde, acrolein and crotonaldehyde (R = CH_3_)] and **h** nitric oxide. Crosslinking agents are shown in *red*

The most simple nitrogen mustard and the first to be used as a chemotherapeutic agent is mechlorethamine (bis(2-chloroethyl)methylamine) (Fig. 1a). Nitrogen mustards are bifunctional alkylating agents, thus their chloroethyl moieties can bind two bases on opposite strands of DNA. They bind guanine N7 forming a monoadduct leading then to binding a second guanine on the opposite strand in the sequence GpNpC (Fig. 1a). Other nitrogen mustards, such as melphalan and chlorambucil, substitute the methyl group with aromatic groups and they can also bind adenine N3 [4]. The generated ICLs, which only account to up to 5 % of the products, cause a distortion of the double helix with an unwinding of 2°–6° and a bend of around 10° per ICL because of the shortening needed to accommodate the N7–N7 bond [5, 6]. Recently, nitrogen mustards have been engineered into activatable prodrugs. These new types of aromatic nitrogen mustards generate ICLs only in the presence of H_2_O_2_ providing a promising tool for the treatment of tumours in a highly oxidative environment [7].

### Mitomycin C

Mitomycin C (MMC) is a natural compound produced by *Streptomyces caespitosus*. It is unable to bind DNA directly but needs to be metabolically reduced beforehand. This need for a reduction step for its activation makes MMC especially fit as a chemotherapeutic agent since the tumour micro-environment is generally hypoxic [8]. After reduction it specifically reacts with the N2 in guanine in the sequence CpG and its complementary strand to form an ICL (Fig. 1b) [8]. However, it can also form monoadducts through the N7 of guanine [9]. ICLs constitute around 15 % of the products (the rest being 50 % monoadducts and 35 % intrastrand crosslinks) [10]. MMC binds to the N2 of guanines through the minor groove and it causes only a minor distortion of the double helix [11].

### Platinum compounds

**Fig. 2:**
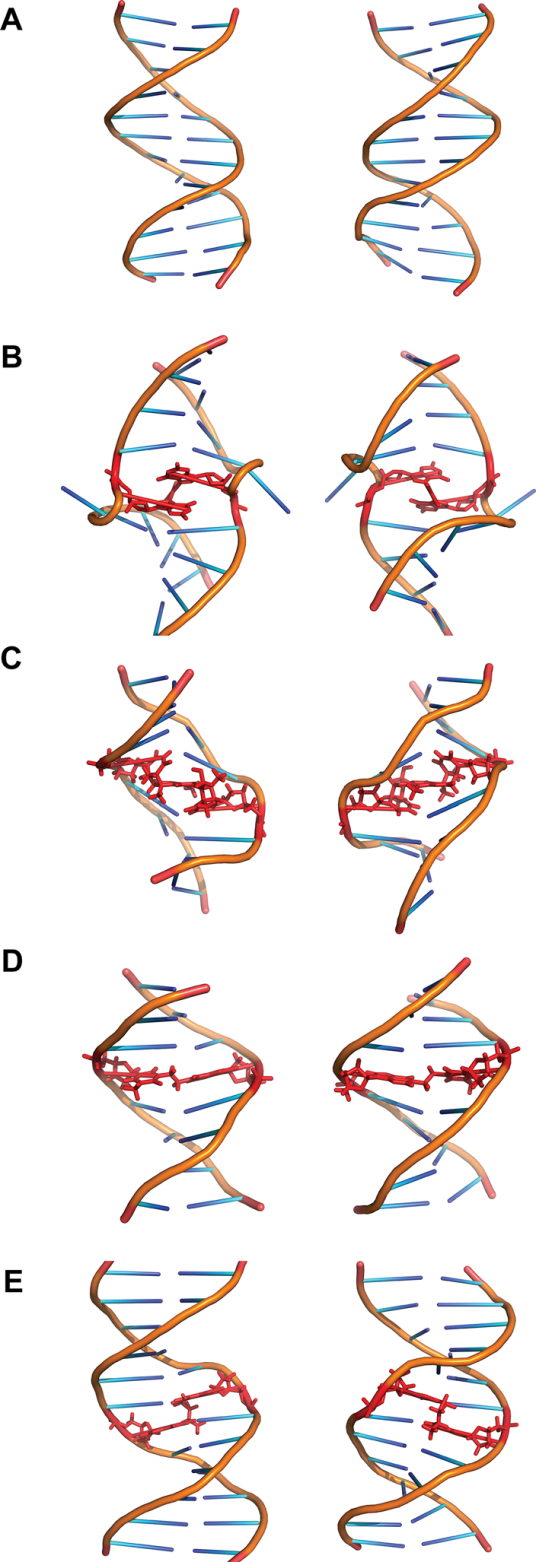
Structures of various ICLs. **a** B-DNA and the ICLs formed by **b** cisplatin, **c** psoralen, **d** BCNU and **e** acetaldehyde and crotonaldehyde viewed from the major groove (*left*) or the minor groove (*right*). The crosslinked bases and the crosslinking agents are shown in *red*. Structures taken from PDB, accession numbers: B-DNA (1-BNA) [140], cisplatin (1A2E) [13], psoralen (204D) [21], BCNU (2MH6) [23], acetaldehyde (2HMD) [29]

Cisplatin [*cis*-diamminedichloroplatinum(II)] was first described as a compound inhibiting bacteria growth in 1965 [12]. It reacts with purine residues to form intrastrand and interstrand crosslinks. The intrastrand crosslinks are formed at sequences GpG and ApG with a preference for the former (65 and 25 % intrastrand crosslinks of total adducts formed, respectively). ICLs are formed with a lower frequency of around 5–8 %. ICLs are formed specifically at GpC sites and bind N7 of guanine (Fig. 1c) [13]. Both crystallographic and NMR structural models have shown cisplatin ICLs to provoke a large distortion in the DNA double helix. These ICLs induce the extrusion of two cytosines in the GC/CG sequence while the platinum locates itself in the minor groove. The double helix suffers an unwinding of 110° and a bent towards the minor groove of 47° (Fig. 2b) [13, 14]. Other platinum compounds that have also been studied and used as chemotherapeutic agents include carboplatin and transplatin. Carboplatin differs from cisplatin in the chloride groups, which are substituted with cyclobutyldicarboxylate. Carboplatin has less reactivity than cisplatin, but it behaves similarly regarding generation of ICLs and their frequency [15]. Transplatin, however, has been shown to induce only minor distortion of the double helix when forming ICLs [16]. Moreover, by substituting the ammine groups in transplatin with the planar bases quinoline or thiazole the frequency of ICLs among the platinated products greatly increased to around 30 % [17].

### Psoralens

Psoralens belong to a family of molecules called furocoumarins. These compounds are produced by at least eight families of plants including Apiaceae and Fabaceae [18]. The planar and hydrophobic nature of these molecules allows them to easily penetrate the cell and intercalate the DNA bases. However, they are unable to form ICLs until irradiated with UVA (ultraviolet light), which induces covalent bonds to thymines on the sequence TpA on opposite strands (Fig. 1d). Psoralens are very effective inducers of ICLs with around 40 % of adducts generated being ICLs. However, a derivative of psoralen, trimethylpsoralen (TMP) can form up to 90 % ICLs [19]. This is due to the incapacity to form intrastrand crosslinks since psoralen must first intercalate the bases on opposing strands of DNA before being photo-activated [20]. The ICLs generated do not bend the DNA double helix and only provoke a minor unwinding of around 25° (Fig. 2c) [21].

### Nitrosoureas

Nitrosoureas such as BCNU (1,3-bis(2-chloroethyl)-1-nitrosourea) are able to react with guanine and cytosine after metabolic activation. Their preferred site for binding is the N7 of guanine, although the O6 of guanine can also be attacked. Through this interaction BCNU can form intrastrand crosslinks between adjacent guanines. ICLs are formed in vitro when BCNU attacks O6 of guanine and N3 of cytosine on opposing strands though these adducts are minor products [22]. Other ICLs formed includes the binding of N1 of guanine with N3 of cytosine, which has been observed in vivo in the treatment of brain cancers (Fig. 1e). The structure of this crosslink has been studied through NMR and was found to be well accommodated in the double helix with very minor alterations (Fig. 2d) [23].

### Diepoxybutane

1,2,3,4-Diepoxybutane (DEB) is a product of the biotransformation of 1,3-butadiene, a contaminating gas produced in the plastic and rubber industry. DEB is a bifunctional alkylating agent and reacts with DNA to produce monoadducts, ICLs, single-strand breaks and also DNA–protein crosslinks. However, in vivo ICLs are the main product responsible for its cytotoxicity [24]. DEB preferentially reacts with N7 and N1 in guanine, although ICLs are formed through N7–N7 at the sequence GpCpC similarly to the nitrogen mustard mechlorethamine (Fig. 1f) [25]. The bridge formed only contains four carbon atoms, which has been hypothesized to produce a major distortion on DNA. Through gel retardation experiments, it has been shown to lead to a bending of around 34° towards the major groove [26].

### Endogenous crosslinking agents

Endogenous ICLs are especially difficult to study. Most evidence of endogenous crosslinking agents comes from in vitro studies or assessment of their mutagenicity [3]. Reactive aldehyde has been thought to be the major endogenous crosslinking agent. One such aldehyde is acetaldehyde. It was shown that acetaldehyde poses similar cellular toxicity to FA-deficient cells compared to other crosslinking agents suggesting that the FA pathway is required for the repair of acetaldehyde-derived damage [2]. Acetaldehyde can be derived from the metabolism of ethanol. It is able to react with guanine on DNA and, after a reduction step, form N2-ethyl-2′-deoxyguanosine. Although this compound is the major adduct formed by acetaldehyde, it cannot form ICLs. Two acetaldehyde molecules can also react with guanine to form 1-N2-propano-2′-deoxyguanosine, though the presence of basic molecules such as histones is needed. This compound is also generated by crotonaldehyde and it can exist in a cyclic or open chain configuration. In the open chain form the free aldehyde group can induce ICLs and DNA–protein crosslinks [27]. These ICLs are generally found in a CpG sequence but DNA–protein crosslinks constitute the main type of modification generated by acetaldehyde (Fig. 1g) [28]. These ICLs induced at CpG sequences by either crotonaldehyde or acetaldehyde are located in the minor groove and do not disturb the Watson–Crick pairing of the bases implicated (Fig. 2e) [29].

Another endogenous source of ICL formation is lipid peroxidation from oxidative stress, a process which is promoted by a fat-rich diet in mice and potentially in humans [30]. Lipid peroxidation leads to the production of malondialdehyde (MDA). MDA can react with guanine, adenine and cytosine though the main ICL produced is between the guanines in the sequence CpG [31]. Other products of lipid peroxidation include unsaturated aldehydes such as acrolein and crotonaldehyde. These can also come from exogenous sources such as cigarette smoke and automobile exhaust [32]. These aldehydes can react with nitrogen bases either through the carbonyl group or the double bond. The conjugate addition is followed by cyclization onto the base to generate a monoadduct. The ICL formed is present in CpG sequences as well and does not disturb the structure of the double helix [32, 33].

Nitric oxide (NO) has also been shown to induce ICLs. It generates ICLs between two guanine residues in the CpG sequence, which are bound by a common N2 amine group (Fig. 1h). This reaction might be favoured by the presence of methylated cytosines [34].

## The Fanconi anemia pathway

Our knowledge of an ICL repair pathway originates from studies of an autosomal recessive disease called Fanconi anemia (FA). FA is a rare genetic disorder with an incidence of 1/200,000–1/400,000 in the general population [35]. FA is characterized by developmental abnormalities and early bone marrow failure, which leads to aplastic anaemia. FA patients are susceptible to various types of cancer, most often acute myelogenous leukaemia (AML). The mechanism behind bone marrow failure in FA is thought to be related to an excessive inflammatory response and apoptosis mediated by tumour necrosis factor α (TNFα), IFNγ and reactive oxygen species (ROS) [36]. To date, 19 FA genes have been identified. Mutation in these genes accounts for 95 % of the FA patients. Patients are sensitive to ICL-forming agents, such as mitomycin C, due to the cellular failure to repair ICLs. There is growing evidence that the symptoms observed in FA patients are also related to this defect in DNA repair. For instance, the bone marrow failure characteristic of FA patients could be originated from a defect in ICL repair in hematopoietic stem cells exposed to endogenous crosslinking agents such as formaldehyde [37]. This defective hematopoiesis leads to cell death, injury and generates an inflammatory response as previously observed, which further enhances bone marrow failure through apoptosis, production of ROS and inhibition of stem cell function [37, 38].

These 19 genes encode proteins, which together with non-FA proteins as well as proteins from other DNA repair pathways, including homologous recombination (HR), nucleotide excision repair (NER) and translesion synthesis (TLS), coordinate the detection and repair of ICLs as well as activation of the cell cycle checkpoint (Table 1) [35, 39]. The 19 FA proteins can be divided into three groups according to their functions in the pathway: the FA core complex, the FANCD2/FANCI complex and the effector proteins (Table 1).

**Table 1:** FA proteins identified to date, their synonyms, size and function

FA protein	Synonym	Size (aa)	Function
FANCA	–	1455	FA core complex
FANCB	–	859	FA core complex
FANCC	–	558	FA core complex
FANCD1	BRCA2	3418	Homologous recombination
FANCD2	–	1451	Essential for the recruitment of downstream effector proteins
FANCE	–	536	FA core complex
FANCF	–	374	FA core complex
FANCG	XRCC9	622	FA core complex
FANCI	–	1328	Essential for the recruitment of downstream effector proteins
FANCJ	BRIP1, BACH1	1249	Homologous recombination, helicase
FANCL	–	380	FA core complex, E3 ubiquitin ligase
FANCM	–	2048	FA core complex, DNA translocase
FANCN	PALB2	1186	Homologous recombination, BRCA2 partner
FANCO	RAD51C	376	Homologous recombination
FANCP	SLX4	1834	Scaffolding protein for nucleases
FANCQ	ERCC4, XPF	916	ERCC1 partner, nuclease
FANCR	RAD51	340	Homologous recombination
FANCS	BRCA1	1863	Homologous recombination, removes CMG
FANCT	UBE2T	197	FANCL partner, E2 conjugating enzyme

Information based on [35, 39]. Although FANCM classification as an FA protein is controversial, it is still traditionally included (please see text and [43, 44])

First, eight FANC proteins (FANCA, FANCB, FANCC, FANCE, FANCF, FANCG, FANCL and FANCM) and three associated proteins (FAAP20, FAAP24 and FAAP100) form the FA core complex. Among other proteins that bind to some components of the core complex as part of the core complex or forming independent complexes, we find BLM (Bloom syndrome helicase), Topo IIIα (topoisomerase IIIα), RPA (replication protein A) and MHF1/2 (histone fold heterodimer) [40–42]. BLM, Topo IIIα and RPA interact with FANCA, FANCC, FANCE, FANCF and FANCG in a complex named BRAFT. This complex potentially plays a role in the FA pathway since BLM deficiency leads to sensitivity to MMC [41, 42]. On the other hand, MHF1/2 bind to FANCM and are recruited to replication forks stalled by ICLs. MHF1/2 are also needed for resistance to ICLs and promote FANCD2 monoubiquitination [40]. However, there is some debate regarding whether FANCM can be considered an FA protein. The controversy arises from the fact that the first FA patient identified with biallelic mutations in *FANCM* also had alterations on *FANCA* [43] and also the observation that individuals with homozygous loss of function of *FANCM* did not display FA symptoms [44]. Despite these observations, FANCM is usually included as an FA protein and a component of the core complex. The FA core complex together with FANCT (UBE2T) [45–47], a ubiquitin-conjugating enzyme, is responsible for monoubiquitination of the FANCD2/FANCI complex. FANCL, an E3 ligase, is the catalytic enzyme carrying out the ubiquitination. While mutations in some of the FA core complex members, such as FANCA, FANCC and FANCG, account for 85 % of the FA patients worldwide, the exact function of these members remains elusive. FANCL and UBE2T are sufficient to monoubiquitinate FANCD2/FANCI complex in vitro [48–50] and, for instance in silkworm; there is an active FA pathway in the absence of the FA core complex [51]. Loss of different FA core complex members causes variable degrees of sensitivity towards crosslinking agents. Some of the FA core complex members are predicted to be entirely helical and have no known conventional domains [52], which makes it difficult to speculate on their molecular functions. Recently, it was shown that a minimal subcomplex containing FANCB, FANCL and FAAP100 is required for robust FANCD2 monoubiquitination in DT40 cells and in vitro [53]. The rest of the FA core complex can be divided into two subcomplexes, FANCA-FANCG-FAAP20 and FANCC-FANCE-FANCF. Their presence facilitates the activity and the recruitment of the whole FA core complex onto DNA [54]. Nonetheless, this function would be redundant with that of the translocase FANCM [55]. The integrity of the FA core complex is also modulated by post-translational modifications, e.g. phosphorylation by ATR/Chk1 and ubiquitination.

Second, the FANCD2/FANCI complex resides at the heart of the FA pathway. It is monoubiquitinated by the FA core complex and is recruited to the ICLs. This is a critical step for the ICL repair. If there is no monoubiquitination, there will be no subsequent repair of the ICL. The function of monoubiquitinated FANCD2/FANCI complex is not fully understood. It is thought to orchestrate the recruitment of the downstream effector proteins to the ICL. In addition to monoubiquitination, the FANCD2/FANCI complex is also regulated by other post-translational modifications that we will discuss extensively in a later section.

Third, FANCD1 (BRCA2), FANCJ (BRIP1), FANCN (PALB2), FANCO (RAD51C), FANCP (SLX4), FANCQ (XPF), FANCR (RAD51) and FANCS (BRCA1) are the effector proteins that contribute to the ICL repair at later stages (Table 1). BRCA1, BRCA2, BRIP1, PALB2, RAD51 and RAD51C have been known for their roles in homologous recombination, which plays an important part in the FA pathway. Mutations in either BRCA1 or BRCA2 lead to higher risk of breast and ovarian cancer [56]. Recently, mutations in other FA genes such as BRIP1, PALB2 and RAD51C have been associated with an intermediate risk of breast cancer [57, 58]. FANCJ was shown to interact with BLM, promoting its stability. This interaction is probably distinct from the BRAFT complex and plays a potential role in the response to replication stress [59, 60]. SLX4 is a nuclease scaffold protein interacting with several nucleases including XPF/ERCC1, MUS81/EME1 and SLX1. However, XPF is thought to be especially important in ICL unhooking.

In addition to the 19 FA proteins and 3 FA associated proteins, there are other non-FA proteins that have been shown to participate in the ICL repair. For example, UHRF1 has been proposed to recognize ICLs in vivo and in vitro [61, 62]. FAN1 (Fanconi-associated nuclease 1) has been shown to be one of the nucleases important for the ICL repair [63–67]. SNM1A is another nuclease that has been demonstrated to participate in the ICL repair [68, 69].

Although the focus of this review is the response to ICLs, alternative roles for the FA proteins are emerging in recent years. There is growing evidence for the role of FA proteins in replication fork protection and recovery after stalling, whether caused by ICLs or other genomic stresses. Monoubiquitinated FANCD2 has been shown to recruit the nuclease FAN1, as well as other FA mediators such as BLM, FANCJ and BRCA2, independently of the core complex, to promote fork recovery and genomic stability [70–72]. Another source of genomic instability are the ultra-fine DNA bridges or UFBs that interlink chromosomes during mitosis. These UFBs are thought to arise from common fragile loci that associate with FANCD2 and FANCI even through mitosis when BLM is also found at the UFBs. These proteins are thought to contribute to the resolution of the UFBs ensuring a correct chromosomal segregation, but the exact mechanism remains unclear [73, 74]. FA proteins have also been associated with the processing of transcription associated DNA: RNA hybrids, also known as R-loops, and the stabilization of replication forks stalled by these structures [75]. Mainly, FANCM was found to resolve R-loops through its translocase activity and, surprisingly, aldehydes were observed to induce R-loops, adding another by-product of their activity to the different adducts already discussed [75].

## Post-translational modifications of the FA proteins

**Fig. 3:**
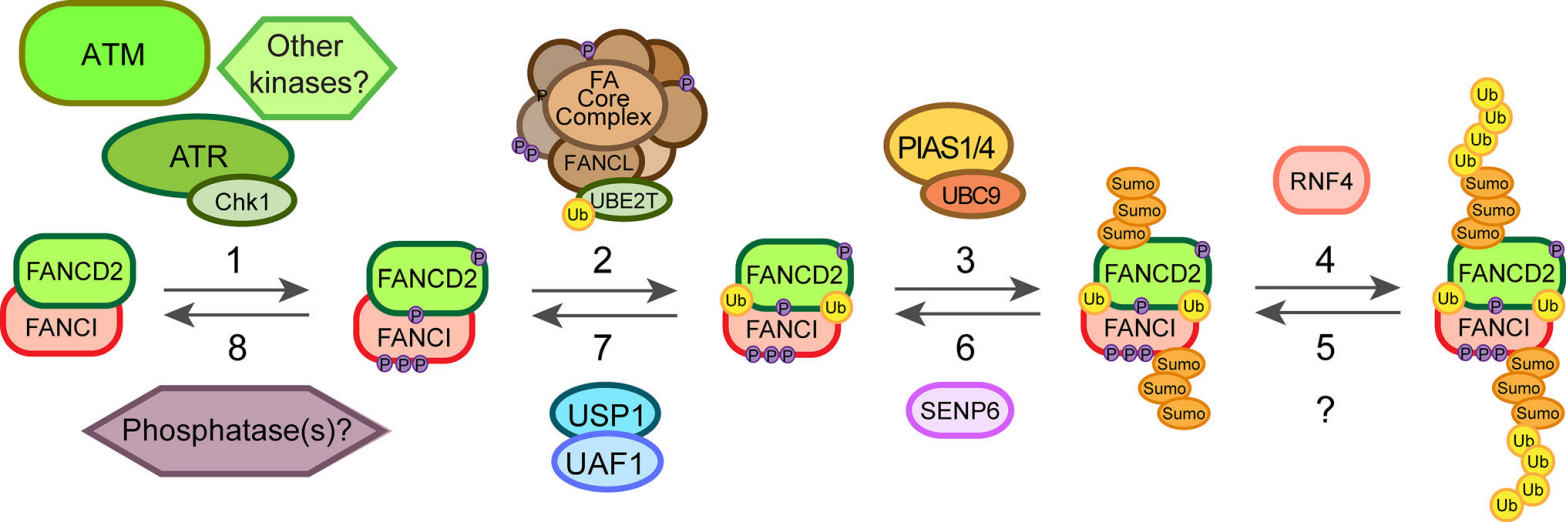
Diagram of the main posttranslational modification events involved in the activation of response to ICLs. Once the ICL is detected it triggers the ATR/Chk1 pathway leading to the phosphorylation of several components of the FA core complex. ATR and potentially other kinases phosphorylate FANCI and FANCD2 (*1*) priming the complex for its monoubiquitination. These phosphorylation events lead then to the monoubiquitination of the FANCD2/FANCI complex by FANCL/UBE2T (*2*), which promotes its recruitment onto chromatin and the action of the effector proteins. On the other hand, the dosage of the FANCD2/FANCI complex on chromatin can be regulated through SUMOylation-dependent polyubiquitination mediated by PIAS1/4, UBC9 and RNF4 (*3*, *4*). Finally, these events can be reversed by the action of a hypothetical deubiquitinase (*5*), SENP6 (*6*), the deubiquitinating enzyme USP1/UAF1 complex (*7*) as well as putative phosphatases (*8*) still unidentified

Repair of an ICL is a highly complex process involving the FA pathway as well as other repair pathways. Post-translational modifications (PTMs) play an essential role in the regulation of this process. Depending on the type of modification, PTMs can cause protein conformation or surface charge changes or could establish new protein–protein interactions that trigger signal transduction or degradation. There are many PTM events in the FA pathway identified over the past decade (Table 2). One of the keystones is the monoubiquitination of FANCD2 that is required for its localization at the ICLs. However, this modification is preceded by several phosphorylation events on different proteins and mainly mediated through ATR/ATM (ataxia telangiectasia mutated) kinases and downstream target kinases Chk1 and Chk2. Additionally, recently light has been shed on the role of SUMOylation in the FA pathway [76, 77] as well as on the termination events that shut off the pathway in a timely manner once the repair has been completed (Fig. 3). It should be noted that many of the PTMs identified so far for the effector proteins implicated in HR have been linked to DSB (double strand break) repair and not to ICLs. However, these mechanisms could play similar roles in response to crosslinking agents as well.

**Table 2:** Summary of the main PTMs of FA proteins and their function in the response and repair of ICLs

FA protein	Site	Post-translational modification	Function
FANCA	S1449	Phosphorylated by ATR	Promotes FANCD2 monoubiquitination specifically after DNA damage [87]
	–	SUMO-mediated by UBC9 and polyubiquitinated by RNF4	Proteasome degradation, pathway termination [77]
FANCE	T346 and S374	Phosphorylated by Chk1	Proteasome degradation, pathway termination [88]
FANCG	K182, K258 and K347	Polyubiquitinated	Interaction with BRCA1 and HR [110]
	S383 and S387	Phosphorylated by Cdc2	Dissociation from chromatin in mitosis, pathway termination [79]
FANCM	S1045	Phosphorylated by ATR	Enhances chromatin localization after DNA damage and S phase [89, 90]
	–	Phosphorylated by Plk1	Degradation of FANCM in M phase and core complex release [92]
FANCD2	S222	Phosphorylated by ATM	Regulation of intra-S-phase checkpoint [78]
	S331	Phosphorylated by Chk1	Interaction with BRCA2, MMC sensitivity [82]
	K561	Monoubiquitinated by FANCL	Enhances chromatin recruitment, interaction with effector proteins [100, 101]
	T691 and S717	Phosphorylated by ATR or ATM	Regulation of intra-S-phase checkpoint, MMC sensitivity [80]
	–	SUMOylated by PIAS1/4 and polyubiquitinated by RNF4	Chromatin dissociation [76]
FANCI	S556, S559, S565, S596 and S617	Phosphorylated by ATR	Required for FANCD2 monoubiquitination [83]
	K563	Monoubiquitinated by FANCL	Maintenance of FANCD2 monoubiquitination [48, 105]
	–	SUMOylated by PIAS1/4 and polyubiquitinated by RNF4	Chromatin dissociation [76]
FANCJ	S990	Phosphorylated probably by Cdks	Regulation of the DNA damage checkpoint [99]
PALB2	K25, K30	Ubiquitinated by KEAP1-CUL3-RBX1	Inhibition of HR during G1 phase [109]
BRCA1	S1497, S1189 and S1191	Phosphorylated by Cdk1	BRCA1 foci formation and DNA damage checkpoint signalling [96]
	S1164 (others)	Phosphorylated by Plk1	BRCA1 foci formation after DSB [95]
	S988	Phosphorylated by Chk2	BRCA1 degradation and dissociation from DSB [97, 98]
	K32 and K1690 (others)	SUMOylated	BRCA1 accumulation on DSB and enhanced ubiquitin ligase activity [111, 112]

### Phosphorylation

The FA pathway relies on several phosphorylation events of different proteins in the core complex and the FANCD2/FANCI heterodimer leading to the monoubiquitination of FANCD2 (Fig. 3). Two kinases are at the centre of the DNA damage response: ATM and ATR that phosphorylate several proteins involved in DNA repair, including other kinases such as Chk1 and Chk2. Although ATM phosphorylates FANCD2 on its S222 in vitro and in human cells, this event is not necessary for its monoubiquitination or ICL repair. Rather, the phosphorylation event regulates the S-phase checkpoint, which inhibits DNA replication after IR treatment (Table 2) [78]. However, ATR was found to be required for FANCD2 monoubiquitination upon MMC or IR treatment. The absence of ATR also abrogated FANCD2 foci formation and led to chromosomal abnormalities in cells derived from a patient with Seckel syndrome [79]. Two sites were identified on FANCD2, T691 and S717, which can be phosphorylated by ATR and ATM both in vitro and in human cells, but could not fully account for the phenotype observed in the absence of ATR. These two sites were not essential for FANCD2 monoubiquitination, though if mutated led to an increase in sensitivity to MMC pointing to a role in ICL repair and also affected the intra-S-phase checkpoint (Table 2) [80]. Other components of the ATR pathway can promote the efficient monoubiquitination of FANCD2 and ICL resistance, mainly RAD9 and RAD17 [81]. It was also found that Chk1 and its partner CLASPIN are necessary for efficient FANCD2 monoubiquitination in response to DNA damage in human cells, but whether this happens downstream or independently of the ATR/RAD17/RAD9 pathway remains unknown [81]. The identification of a new phosphorylation site on FANCD2, S331, shed more light on this problem. The lack of phosphorylation on this site also led to sensitivity to MMC and disrupted the interaction with BRCA2 (FANCD1) in human cells. This site is phosphorylated by Chk1 both in vitro and in vivo, which could be a potential explanation for the role of the ATR pathway through Chk1 in promoting FANCD2 monoubiquitination and ICL repair (Table 2) [82].

Phosphorylation of FANCI was also found to be important for FANCD2 monoubiquitination and foci formation. Several conserved sites (at least six) on FANCI with the motif Ser/Thr-Gln predicted to be phosphorylated by ATM or ATR were responsible for the observations in chicken DT40 cells. This phosphorylation event could be a switch for the monoubiquitination and recruitment of FANCD2 onto DNA. However, the monoubiquitination of FANCI was dispensable for monoubiquitination and recruitment of FANCD2 to ICLs [83]. Further evidence for the role of ATR in the response to ICLs came from experiments with chicken DT40 cells lacking expression of ATRIP, an ATR interacting partner needed for its activation. In this case, there was also a reduction in FANCD2 and FANCI monoubiquitination as well as in FANCI phosphorylation after MMC treatment. FANCI was phosphorylated by ATR in vitro and the reaction was enhanced by the presence of FANCD2 and the core complex [84].

Several components of the core complex also undergo phosphorylation although with different effects on the FA pathway. FANCG, for example, is phosphorylated during mitosis in human cells and this was related to the dissociation of the core complex from chromatin once the repair is completed [85]. Later, two residues were identified as responsible for this event, S383 and S387. S387 was phosphorylated (in vitro and in human cells) by Cdc2, which associates with the core complex in mitosis (Table 2) [86].

A more critical role in the FA pathway and ICL repair was found for the phosphorylation of FANCA on S1449. Phosphorylation on this site occurred specifically after DNA damage and not during unperturbed S-phase, unlike FANCD2 monoubiquitination and FANCG phosphorylation, which happen also during S-phase. Lack of phosphorylation on this site reduced FANCD2 monoubiquitination and led to partial sensitivity to MMC. ATR phosphorylated S1449 in vitro and was necessary for the phosphorylation in human cells (Table 2) [87].

Another component of the core complex, FANCE, is also phosphorylated after DNA damage. Chk1 phosphorylates FANCE on T346 and S374 both in vitro and in human cell lines (Table 2) [88]. However, this event is independent of FANCD2 phosphorylation and foci formation. The fact that FANCE phosphorylation was necessary to fully complement FANCE-deficient cells suggests it is still an important event in the FA pathway and ICL repair. FANCE phosphorylation promotes its degradation, thus it was proposed to play a role in the termination of the pathway to complete the repair [88].

Recruitment of the core complex to DNA is usually attributed to FANCM and its phosphorylation could play a critical role in the process. Studies in cell-free *Xenopus* egg extracts showed that FANCM is hyperphosphorylated during S-phase as well as after DNA damage and this enhances its chromatin localization. ATR and ATM regulate this process. Surprisingly, these events were shown to be favoured by the presence of FANCD2, pointing to a positive feedback-loop taking place (Table 2) [89, 90]. Similar results were obtained in human cell lines where S1045 on FANCM was found to be a target for ATR and necessary for its localization on ICLs as well as the activation of the G_2_-M checkpoint [90]. There is evidence for a role of ATR physically mediating the recruitment of FANCM to the damaged DNA during replication in human cells. ATR/ATRIP is recruited to stalled replication forks through interaction with RPA, which then interacts with HCLK2. HCLK2 can then recruit the heterodimer formed by FANCM and FAAP24, providing a potential mechanism for the recruitment of the core complex in an ATR-checkpoint signalling dependent manner [91]. On the other hand, when the cell enters M-phase FANCM is hyperphosphorylated and degraded, thus promoting the dissociation of the core complex from chromatin. This phosphorylation-dependent degradation was mediated by β-TRCP and Plk1 (Polo-like kinase 1) in human cells [92].

Among the effector proteins, phosphorylation of those implicated in HR is well established in response to IR and therefore, in DSB repair. However, given that these modifications may also play a role in ICL repair we will discuss some of them (Table 2). Phosphorylation of BRCA1 by ATM was one of the first modifications found that played a role in DSB repair [93]. Later, BRCA1, BRCA2 and PALB2 together with other proteins already discussed (FANCD2, FANCI) were identified as substrates for ATR and ATM in response to DSBs as part of a complex protein network [94]. BRCA1 is a key regulator of HR and several kinases have been linked to its function and regulation. Cdk1 and Plk1 phosphorylate BRCA1, probably in a sequential way, promoting BRCA1 foci formation following DSB in human cells [95, 96]. On the other hand, Chk2 phosphorylation of BRCA1 leads to its degradation and dissociation form DSB in human cells. This allows the nuclease MRE11 to be recruited so that end resection and HR can proceed during S/G2 phase [97, 98]. The BRCT domain of BRCA1 has been characterized as a phosphoprotein-binding domain and one of its binding partners is FANCJ. Phosphorylation of FANCJ on S990 during S/G2 phase was shown to be essential for the interaction with BRCA1 in human cells, controlling the cell cycle as part of the DNA damage checkpoint [99].

### Ubiquitination

A central step in the FA pathway is the monoubiquitination of FANCD2 on Lys561 (human) that ensures its recruitment to damaged DNA as well as its interaction with other effector proteins such as BRCA1 (FANCS) (Table 2) [100]. The E3 ubiquitin ligase catalysing this step was found to be FANCL, a member of the core complex [101]. FANCL works together with the E2 conjugating enzyme UBE2T (FANCT) to monoubiquitinate FANCD2 (Fig. 3). UBE2T can also monoubiquitinate itself on K91 decreasing its own activity as a potential regulatory mechanism [102]. FANCL contains three domains: an N-terminal E2-like fold (ELF) domain, a central double RWD domain and a C-terminal RING domain. The RING domain binds to UBE2T while the RWD domain binds to FANCD2 [103]. The ELF domain interacts with ubiquitin and is important for the monoubiquitination of FANCD2 upon DNA damage in chicken DT40 cells (though not in vitro) [104]. The binding partner of FANCD2, FANCI, is also monoubiquitinated in vivo, and is required for the monoubiquitination of FANCD2 and restricts it to K561 [48, 105]. Furthermore, the presence of DNA greatly enhances FANCD2 monoubiquitination but only in the presence of FANCI in vitro, suggesting that the monoubiquitination in vivo occurs on the DNA and when FANCD2 and FANCI are in complex [49]. Finally, FANCD2 is deubiquitinated by the USP1/UAF1 deubiquitinating enzyme complex (Fig. 3) [106, 107].

Ubiquitination also plays a critical role in an alternative model for the recruitment of the core complex. In this study in human cells, RNF8 together with UBC13 promotes K63 polyubiquitination of histone H2A in response to DNA damage and this polyubiquitin is recognized by FAAP20 bringing the core complex onto damaged DNA [108]. In fact, RNF8 and FAAP20 were needed for efficient FANCD2 monoubiquitination after MMC treatment and, thus, for efficient ICL repair [108].

Ubiquitination has also been linked to the regulation of effector proteins implicated in HR. PALB2 and BRCA1 interaction is required for HR during G1 phase. However, PALB2 ubiquitination on its BRCA1 binding motif abrogates this interaction in 293T cells after IR (Table 2) [109].

Recently, the polyubiquitination of FANCG via K63 linkage has been found to mediate its interaction with BRCA1 and play an important role in HR in ICL repair in human cells [110]. Three potential target lysines for this process were identified: K182, K258 and K347 (Table 2) [110].

### SUMOylation

Alongside the ubiquitination and phosphorylation events in the FA pathway, several SUMOylation events have started to be discovered in recent years. SUMOylation, therefore, provides a further step of regulation of the already complex cellular response to ICLs. As discussed before, FANCD2 and FANCI form a heterodimer that is both phosphorylated and monoubiquitinated in order to appear on ICLs. A subpopulation of FANCD2 and FANCI is also SUMOylated in response to DNA damage. This SUMOylation is performed by PIAS1/4 and UBC9 on the chromatin-bound complex in human cell lines while it can be reversed by SENP6 (Table 2) [76]. SUMOylated FANCD2/FANCI can then bind RNF4 which polyubiquitinates the complex. This polyubiquitinated complex then interacts with DVC1-p97 promoting its dissociation from chromatin (Fig. 3). Therefore, this mechanism could control the dosage of FANCD2/FANCI on the chromatin avoiding further recruitment of nucleases to DNA and allowing for a dynamic regulation of the pathway [76].

The study of a patient-derived FANCA mutation identified in the clinic has led to the discovery of a regulatory mechanism through its SUMOylation-dependent polyubiquitination and degradation. This mutant form, FANCA-I939S, failed to interact with FAAP20 and this led to its SUMOylation by UBC9, increased polyubiquitination by RNF4 and degradation via the proteasome (Table 2) [77].

SUMOylation of BRCA1 by SUMO2/3 in response to DSB has also been described both in vitro and in vivo in mammalian cell lines [111, 112]. SUMOylation of BRCA1 at its RING and BRCT domains (K32 and K1690, respectively) promotes the binding to other proteins through SIMs (SUMO-interacting motifs), which in this case would enhance accumulation on DNA and its ubiquitin ligase activity [111, 112].

These examples could illustrate a more general mechanism for SUMO signalling as already shown for DNA double-strand break repair [113]. In this case, SUMO modifications target several proteins in a group, such as the core complex or the FANCD2/FANCI complex, thereby promoting interactions through the SUMO-SIMs of the components synergistically. At a later stage, this leads to their polyubiquitination and degradation to ensure the termination of the repair and the progression of the cell cycle.

## Recognition of the ICL

**Fig. 4:**
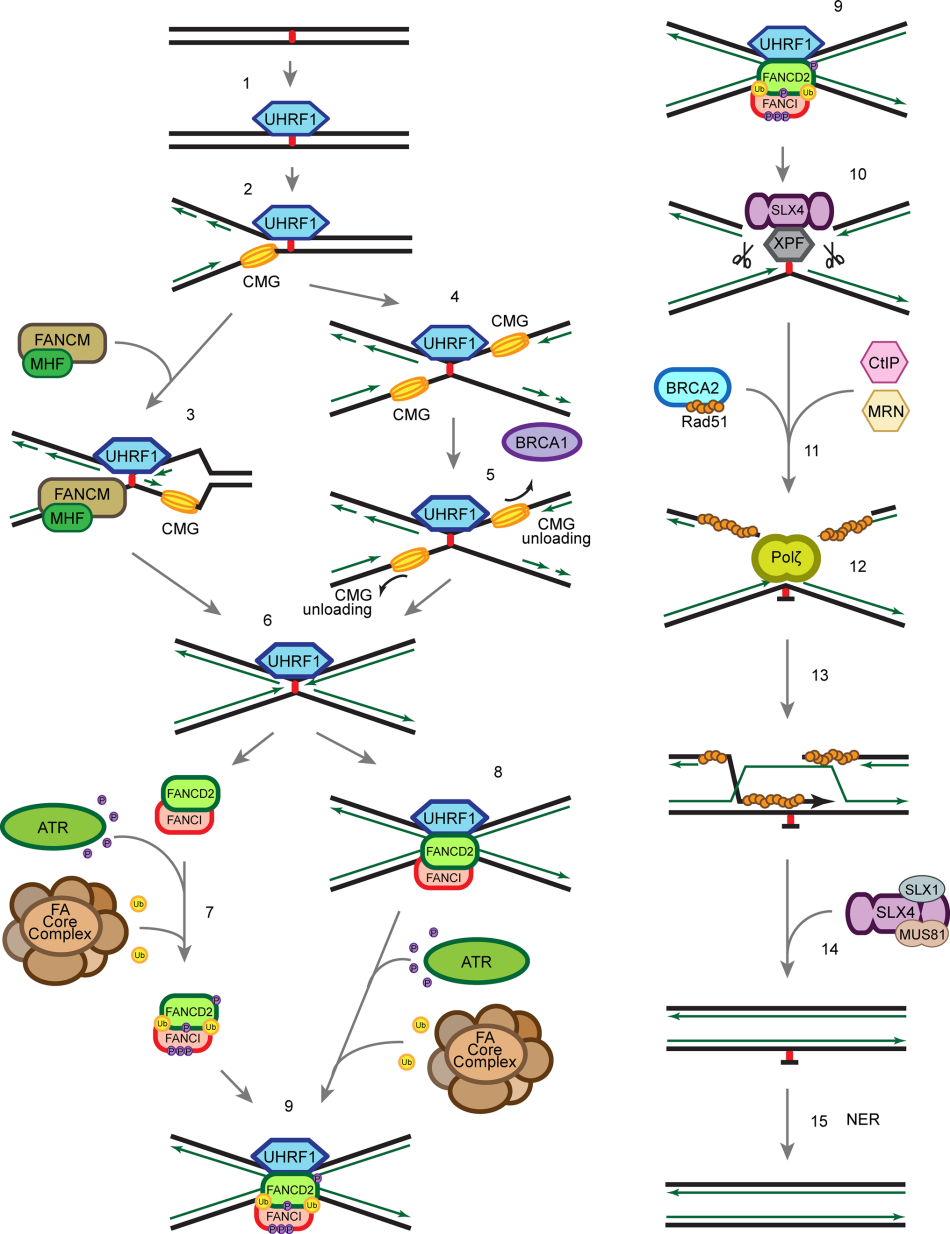
Schematic of ICL repair. *1* UHRF1 is recruited to ICLs through its SRA domain shortly after ICLs are formed in the cell. *2* Single replication fork arrives at the ICL. *3* FANCM/MHF complex mediates the traverse of the replication machinery through the ICL, which allows the replication fork to proceed, and leaves the ICL for later repair. *4*, *5* Alternatively, BRCA1 (FANCS) facilitates the unloading of the CMG helicase complex when the second replication fork arrives at the ICL. *6* The replicative polymerase proceeds to the −1 position of the ICL, which leaves an X-shaped structure similar to the traverse mechanism. *7* ATR phosphorylates FANCD2/FANCI complex at multiple sites and FA core complex monoubiquitinates FANCD2/FANCI complex at K561 and K523, respectively. *8* FANCD2/FANCI is recruited to the ICL at the replication fork. *9*, *10* Ubiquitinated FANCD2/FANCI complex recruits SLX4/XPF to ICL to unhook the ICL. *11* CtIP and the MRN complex resect the double-strand break ends generated by the incision in the previous step, and BRCA2 facilitates Rad51 filament formation on the ssDNA generated by the resection. *12* Polζ polymerizes new strand of DNA through the unhooked ICL. *13* Rad51 facilitates the strand invasion, which allows extension of the other strand. *14* SLX4 and nucleases resolve the double Holliday junction. *15* NER repair proteins remove the damaged nucleotide

When ICLs occur in the cells, the UHRF1 protein is recruited to sites of damage within seconds [61, 62] (Fig. 4, step 1). UHRF1 recognizes ICLs through its SET and RING finger associated (SRA) domain, which was previously known for its role in recognizing hemi-methylated DNA and subsequent recruitment of DNMT1 to maintain the methylation signature in mammalian cells [114–117]. The affinities of UHRF1 to hemi-methylated DNA and to ICLs are similar, suggesting that UHRF1 could interact with hemi-methylated DNA and ICLs through related mechanisms. The recruitment of UHRF1 precedes and is required for proper recruitment of FANCD2 to ICLs [61]. There is about 10 min time lag between UHRF1 and FANCD2 recruitment to ICLs, which leaves room for speculation that recruitment of other proteins or PTM events might take place within this time frame. The exact mechanism of how UHRF1 facilitates FANCD2 recruitment or subsequent repair is still unclear, but might entail a direct protein–protein interaction. It has also been suggested that UHRF1 plays a role as a nuclease scaffold [62]. It is possible that the rapid recruitment of UHRF1 to the ICLs primes the lesion for FA mediated accurate repair later. Due to the diversity in structures of different ICLs (Figs. 1, 2), it is possible that other ICL sensor proteins exist in addition to UHRF1.

## Canonical FA pathway coordinated ICL repair

It is generally believed that the FA pathway is active solely in the S-phase and that the majority of ICLs are repaired in a replication-dependent manner [118]. The repair of ICLs requires coordination of several different DNA damage repair pathways including NER, HR and TLS. It is thought that the FANCD2/FANCI complex and the FA pathway is at the centre orchestrating the order of various events to resolve ICLs. Notably, it has been suggested that the FA pathway antagonizes the NHEJ pathway [119–121], which further emphasizes the importance of the FANCD2/FANCI complex.

There are several models of the replication-coupled ICL repair (Fig. 4). One possibility is that the fork undergoes FANCM/MHF complex-mediated traverse of the ICL, which is independent of the rest of the FA core complex and FANCD2/FANCI complex [19] (Fig. 4, step 3). The ICL traverse mechanism leaves behind the ICL, which has an X-shaped structure similar to that of a stalled replication fork. Due to the structure similarity, it has been implied that it is subsequently repaired by the canonical ICL repair mechanism.

Alternatively, when the replication fork collides with an ICL, the CMG helicase first undergoes BRCA1 (FANCS)-mediated unloading to allow the replicative polymerase ε to approach the ICL [122] (Fig. 4, step 5). The FANCD2/FANCI complex is then recruited to the arrested fork (Fig. 4, step 8), and monoubiquitinated by the FA core complex [123] (Fig. 4, step 9). Whether the FANCD2/FANCI complex is monoubiquitinated prior to recruitment, or on chromatin, remains elusive (Fig. 4, step 7). The K561R mutation of FANCD2 abrogates monoubiquitination and chromatin recruitment of FANCD2/FANCI complex and sensitizes cells to crosslinking agents, [78, 124]. However, it is also possible that the monoubiquitination of FANCD2/FANCI complex is critical for its retention at the ICL rather than the actual recruitment to the ICL. Recent studies show that DNA stimulates the ubiquitination of FANCD2/FANCI in vitro [49, 53, 125], which implies that the interaction between the FANCD2/FANCI complex and DNA may contribute to the regulation of its monoubiquitination in vivo. The monoubiquitinated FANCD2/FANCI complex recruits the XPF/ERCC1/SLX4 nuclease complex (Fig. 4, step 10) and the nucleases carry out incisions at the ICL to unhook the crosslinked bases [126–128]. The TLS polymerase Rev1–polζ complex, which can accommodate bulky DNA substrates, is subsequently recruited to the lesion to carry out insertion through the unhooked ICL base pair (Fig. 4, step 12) [129]. Interestingly, the FA core complex, but not the FANCD2/FANCI complex, regulates recruitment of the Rev1–polζ complex [129]. The double-strand break generated by the incision is resected by CtIP, which is recruited through monoubiquitinated FANCD2 [130, 131], potentially together with the MRN (MRE11-RAD50-NBS1) complex and the nucleases EXO1 and DNA2. MRE11 has been shown to interact with FANCJ, which potentially regulates its nuclease activity ensuring a correct end resection [132]. The DSB is, then, repaired by RAD51-mediated homologous recombination (Fig. 4, step 13) [133]. To complete the repair of the ICL, the unhooked lesion needs to be removed. It is generally thought that the NER pathway is involved in the process (Fig. 4, step 15) [134, 135], though the precise molecular mechanism remains elusive.

## Alternative ICL repair pathway in G1

In addition to replication-coupled ICL repair, there is increasing evidence for the existence of a replication-independent ICL repair mechanism. Although it has been understudied, replication-independent ICL repair may play a significant role in maintaining genome integrity in non-cycling cells, e.g. neurons.

It has been shown that ICLs can be removed via the NER pathway independently of replication [136–139]. NER proteins XPA, XPB, XPC and XPF are recruited to psoralen/UVA induced ICLs and monoadducts within minutes in the G1-phase of the cell cycle and this ICL repair process is dependent on Polζ and XPC [137, 138]. It has also been shown that transcription coupled-NER (TC-NER) is crucial for cisplatin-induced ICL repair [136, 139].

The detailed molecular mechanism of the replication-independent ICL repair is still unclear. It is likely that in the absence of arrested replication forks, the activation of ICL repair relies on the proteins recognizing the distorted DNA helix and/or the collision of the RNA transcription machinery with an ICL. There are some contradictions in the literature whether XPC is involved in the replication-independent repair. The ICL-forming agents used in the studies mentioned above differ from study to study, but are still generally considered to pose similar replicative stress. As discussed previously, these crosslinking agents create structurally distinct ICLs, which could give rise to the discrepancies observed.

## Conclusion

Genetic and biochemical studies carried out by various groups have advanced our understanding of ICL repair for the past decade and beyond. 19 FA genes have been identified and the corresponding proteins cooperate with other proteins in HR, NHEJ, TLS and NER to resolve ICLs. However, the more we have learnt, the more questions have arisen. For instance, UHRF1 has been shown to sense ICLs induced by TMP/UVA in vivo. Besides, the recruitment of UHRF1 to ICLs precedes and is required for FANCD2 recruitment. Nonetheless, it is unclear how UHRF1 contributes to the regulation of FANCD2, whether directly or indirectly. Also, it has been well accepted that the FANCD2/FANCI complex is monoubiquitinated prior to its recruitment to ICLs. However, in the crystal structure of the mouse FANCD2/FANCI complex, the ubiquitination sites are embedded in the heterodimer interface. Recent studies show that DNA stimulates the in vitro ubiquitination of the FANCD2/FANCI complex [49, 125], suggesting that the interaction of the complex with DNA might be required for, and thus precede, the ubiquitination event. Such a mechanism could potentially involve a conformational change of the complex upon interaction with DNA, in turn stimulating the ubiquitination, perhaps by allowing the FA core complex access to the target lysines. One of the common features of the FA proteins is the lack of predictable domains, which otherwise could help towards deciphering their exact functions. More structural studies of proteins involved in ICL repair will be required to achieve a full mechanistic understanding of this complicated process. Finally, different crosslinking agents have been thought to cause similar cellular toxicities and the resulting ICLs would be sensed and resolved through the same pathway. However, the nature of the ICLs induced by different crosslinking agents is different. We may need to re-think how we depict the pathways resolving ICLs, which could be more complicated than we currently imagine. For instance, ICLs induced by MMC and TMP/UVA cause a minor distortion of the DNA double helix and can be recognized by XPC and UHRF1, respectively [61, 137]. On the other hand, ICLs caused by cisplatin cause a major distortion of DNA, which may require other sensor proteins or collision of the replication and transcription machineries with the ICL to activate the signal cascade. Thus, different ICLs may lead to activation of different, though related, repair mechanisms.

## Abbreviations

ATM: Ataxia telangiectasia mutated
ATR: ATM and Rad3-related
AML: Acute myelogenous leukaemia
BCNU: 1,3-Bis(2-chloroethyl)-1-nitrosourea
DDR: DNA damage response
DEB: 1,2,3,4-Diepoxybutane
DSB: Double-strand break
FA: Fanconi anemia
HR: Homologous recombination
ICL: Interstrand crosslink
IR: Ionizing radiation
MDA: Malondialdehyde
MMC: Mitomycin C
NER: Nucleotide excision repair
NHEJ: Non-homologous end joining
NO: Nitric oxide
PTM: Post-translational modification
ROS: Reactive oxygen species
RPA: Replication protein A
SUMO: Small ubiquitin-like modifier
TLS: Translesion synthesis
TMP: Trimethylpsoralen
TNF-α: Tumour necrosis factor-α
UAF1: USP1 associated factor 1
UHRF1: Ubiquitin-like containing PHD and RING finger domains 1
USP1: Ubiquitin-specific peptidase 1
